# Retraction: Rolot, M.; O’Sullivan, T.E. Living with Yourself: Innate Lymphoid Cell Immunometabolism. *Cells* 2020, *9*, 334

**DOI:** 10.3390/cells11162555

**Published:** 2022-08-17

**Authors:** Marion Rolot, Timothy E. O’Sullivan

**Affiliations:** Department of Microbiology, Immunology, and Molecular Genetics, David Geffen School of Medicine at UCLA, Los Angeles, CA 900953, USA

The journal retracts the article, “Rolot, M. and O’Sullivan, T. E. Living with Yourself: Innate lymphoid Cell Immunometabolism. *Cells* 2020, *9*, 334” [1] cited above.

Following publication, concerns were brought to the attention of the journal regarding overlap with a previously published article [2].

Adhering to our complaints procedure, an investigation was conducted that confirmed the overlap, and the article is therefore retracted. The authors did not respond to correspondence about the retraction notice.

This retraction was approved by the Editor-in-Chief of the journal *Cells.*
